# Time-controlled adaptive ventilation (TCAV) accelerates simulated mucus clearance via increased expiratory flow rate

**DOI:** 10.1186/s40635-019-0250-5

**Published:** 2019-05-16

**Authors:** Melissa Mahajan, David DiStefano, Joshua Satalin, Penny Andrews, Hassan al-Khalisy, Sarah Baker, Louis A. Gatto, Gary F. Nieman, Nader M. Habashi

**Affiliations:** 1 Department of Surgery, SUNY Upstate Medical University, 750 East Adams St., 766 Irving Avenue, Syracuse, NY 13210 USA; 2 Department of Trauma Critical Care Medicine, R Adams Cowley Shock Trauma Center, University of Maryland School of Medicine, 22 S. Greene Street, Baltimore, MD 21201 USA; 3 Department of Biological Sciences, SUNY Cortland, 22 Graham Avenue, Cortland, NY 13045 USA

**Keywords:** Airway pressure release ventilation (APRV) mode, Expiratory flow rate, Mucus, Time-controlled adaptive ventilation (TCAV), Ventilator-associated pneumonia (VAP), ARDSnet ventilation, Volume assist control (VAC) mode, Mucus removal

## Abstract

**Background:**

Ventilator-associated pneumonia (VAP) is the most common nosocomial infection in intensive care units. Distal airway mucus clearance has been shown to reduce VAP incidence. Studies suggest that mucus clearance is enhanced when the rate of expiratory flow is greater than inspiratory flow. The time-controlled adaptive ventilation (TCAV) protocol using the airway pressure release ventilation (APRV) mode has a significantly increased expiratory relative to inspiratory flow rate, as compared with the Acute Respiratory Distress Syndrome Network (ARDSnet) protocol using the conventional ventilation mode of volume assist control (VAC). We hypothesized the TCAV protocol would be superior to the ARDSnet protocol at clearing mucus by a mechanism of net flow in the expiratory direction.

**Methods:**

Preserved pig lungs fitted with an endotracheal tube (ETT) were used as a model to study the effect of multiple combinations of peak inspiratory (I_PF_) and peak expiratory flow rate (E_PF_) on simulated mucus movement within the ETT. Mechanical ventilation was randomized into 6 groups (*n* = 10 runs/group): group 1—TCAV protocol settings with an end-expiratory pressure (P_Low_) of 0 cmH_2_O and P_High_ 25 cmH_2_O, group 2—modified TCAV protocol with increased P_Low_ 5 cmH_2_O and P_High_ 25 cmH_2_O, group 3—modified TCAV with P_Low_ 10 cmH_2_O and P_High_ 25 cmH_2_O, group 4—ARDSnet protocol using low tidal volume (LTV) and PEEP 0 cmH_2_O, group 5—ARDSnet protocol using LTV and PEEP 10 cmH_2_O, and group 6—ARDSnet protocol using LTV and PEEP 20 cmH_2_O. PEEP of ARDSnet is analogous to P_Low_ of TCAV. Proximal (towards the ventilator) mucus movement distance was recorded after 1 min of ventilation in each group.

**Results:**

The TCAV protocol groups 1, 2, and 3 generated significantly greater peak expiratory flow (E_PF_ 51.3 L/min, 46.8 L/min, 36.8 L/min, respectively) as compared to the ARDSnet protocol groups 4, 5, and 6 (32.9 L/min, 23.5 L/min, and 23.2 L/min, respectively) (*p* < 0.001). The TCAV groups also demonstrated the greatest proximal mucus movement (7.95 cm/min, 5.8 cm/min, 1.9 cm/min) (*p* < 0.01). All ARDSnet protocol groups (4–6) had zero proximal mucus movement (0 cm/min).

**Conclusions:**

The TCAV protocol groups promoted the greatest proximal movement of simulated mucus as compared to the ARDSnet protocol groups in this excised lung model. The TCAV protocol settings resulted in the highest E_PF_ and the greatest proximal movement of mucus. Increasing P_Low_ reduced proximal mucus movement. We speculate that proximal mucus movement is driven by E_PF_ when E_PF_ is greater than I_PF_, creating a net force in the proximal direction.

## Background

Despite advances in critical care medicine, VAP remains a prevalent and difficult problem in ICUs [1]. Although difficult to diagnose, the most recent practice guidelines from the American Thoracic Society and Infectious Diseases Society of America define VAP as pneumonia occurring in a patient who has been mechanically ventilated for more than 48 hours. The diagnosis is suggested by the development of a new or progressive lung infiltrate with associated signs and symptoms of infection (new onset fever, purulent sputum, leukocytosis, tachycardia, decreased oxygenation status) and is confirmed by laboratory detection of the pathogen [2].

Retention of respiratory mucus in the distal airways has been implicated as a pathogenic mechanism of VAP; it impairs immunological responses, alters normal respiratory physiology, and is highly associated with the development of pneumonia [3–6]. Mucus clearance is enhanced with ventilator settings in which the expiratory (outward) flow rate is greater than the inspiratory flow rate. The mechanism of this mucus movement is the net shear force of the airflow on the mucus in the outward direction (two-phase gas-liquid flow mechanism) [4, 7]. Benjamin et al. demonstrated that mechanical ventilation set to inverse ratio ventilation (IRV), whereby expiratory flow exceeded inspiratory flow, promoted mucus clearance in a sheep model [7]. We postulate that a mechanical ventilation strategy with a rapid expiratory as compared to inspiratory flow rate will facilitate respiratory mucus clearance similar to IRV.

Our group has developed a time-controlled adaptive ventilation (TCAV) protocol that uses the airway pressure release ventilation (APRV) mode [8, 9]. APRV is a pressure control ventilation mode characterized by a prolonged time at the inspiratory pressure and a short expiratory release. APRV set with the TCAV protocol is characterized by a significantly greater expiratory flow compared to inspiratory flow, which differs from conventional low tidal volume (LTV) ventilation. The aim of our study was to evaluate the effect of the TCAV protocol on the movement of mucus in an excised porcine lung model fitted with an endotracheal tube (ETT). We hypothesized that the increased expiratory flow rate using the TCAV protocol would cause proximal mucus movement due to a net force in the expiratory direction, improving mucus clearance from the respiratory system.

## Methods

**Fig. 1:**
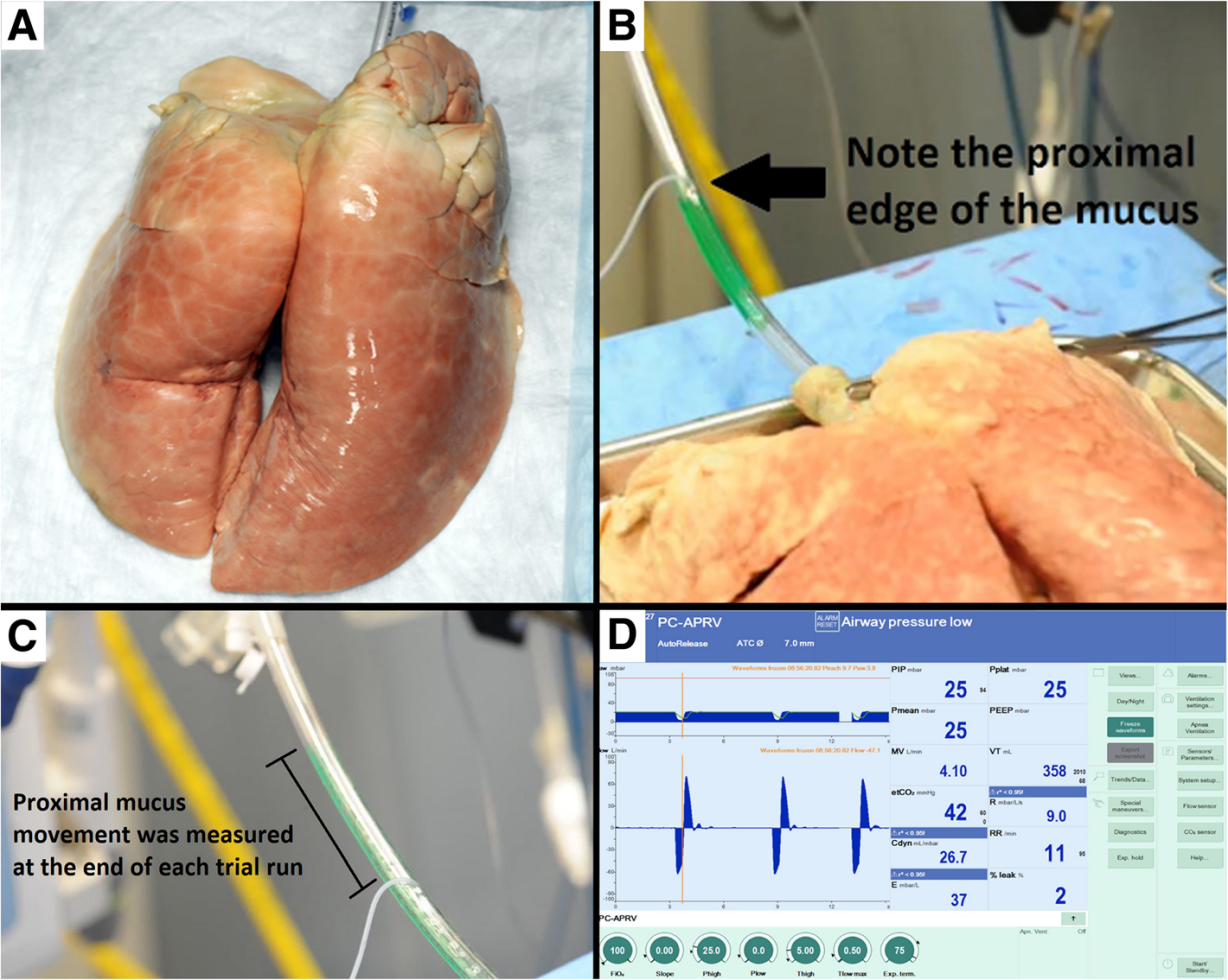
**a** Excised, preserved porcine lungs (Nasco BioQuest® Inflatable Lungs Kit, product number LS03765U). **b** Simulated mucus (3 mL) installed into the middle of the ETT. The proximal edge of the mucus aliquot was marked on the outside of the ETT with a marking pen prior to initiation of mechanical ventilation for each trial run. **c** At the end of each run, the proximal edge of the mucus aliquot was measured again. From these two measurements, the proximal mucus movement was calculated. **d** Ventilator waveforms and settings according to the standard TCAV protocol [8]. The top left graph shows the typical APRV pressure waveform, with a long inspiratory duration followed by a quick expiratory release. The bottom left graph shows the rapid flows generated by the specific TCAV breath

**Fig. 2:**
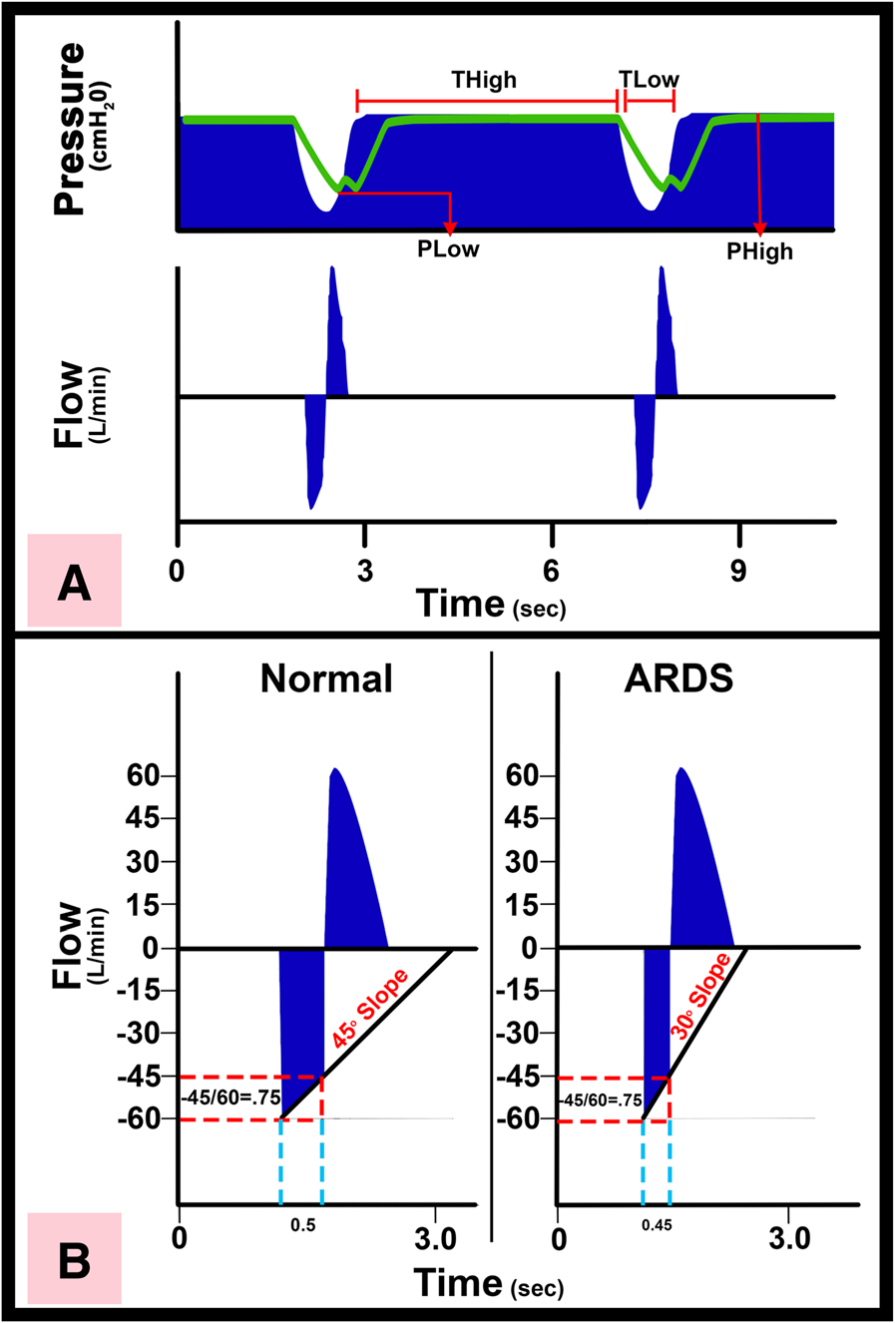
Method of setting time-controlled adaptive ventilation, a protocol using APRV mode with specific settings. **a** Typical time-controlled adaptive ventilation (TCAV, previously referred to as personalized airway pressure release ventilation or P-APRV) airway pressure and flow curves. Correctly set TCAV has a very brief release phase (T_Low_—time at low pressure) and CPAP phase (T_High_—time at high pressure) [8]. The T_High_ is ~ 90% of each breath. The two other TCAV settings are the pressure at inspiration (P_High_) and at expiration (P_Low_), which is always programmed as 0 cmH_2_O. T_Low_ is sufficiently brief such that end-expiratory pressure (P_Low_) never reaches 0 cmH_2_O measured by the tracheal pressure (green line). **b** Alveolar stability is maintained by adaptively adjusting the expiratory duration as directed by the expiratory flow curve. The rate of lung collapse is seen in the normal (slope 45°) and acutely injured lung (ARDS, slope 30°). ARDS causes a more rapid lung collapse due to decreased lung compliance. Our studies have shown that if the end-expiratory flow (E_FT_;− 45 L/min) to the peak expiratory flow (E_PF_;− 60 L/min) ratio is equal to 0.75, the resultant T_Low_ (0.5 s) is sufficient to stabilize alveoli [12, 13]. The lung with ARDS collapses more rapidly such that the E_FT_/E_PF_ ratio of 75% identifies an expiratory duration of 0.45 s as necessary to stabilize alveoli. Figure and figure legend reproduced and modified from Jain et al. 2016 with permission [14]

One set of preserved pig lungs (Nasco BioQuest® Inflatable Lungs Kit, product number LS03765U) fitted with a size 7.0 ETT were repeatedly subjected to multiple combinations of inspiratory and expiratory flow rates using two ventilation protocols, and the movement of simulated mucus was measured (Fig. 1a). The ETT was connected to the lung model and ventilator at an angle of 30 degrees above parallel. Guar gum concentrations of 0.1–1.5% *w*/*v* have been shown to model the rheological properties of human respiratory mucus [10]. We chose a guar gum concentration of 1%, which falls within this range (1 g guar gum/100 mL water, green food coloring). Guar gum was instilled in 3 mL aliquots into the middle of the ETT, and the proximal (toward the ventilator) edge of the mucus aliquot was marked (Fig. 1b). The impact of multiple mechanical ventilation strategies on mucus movement over 1 min was tested (Tables 1 and 2). Groups 1–3 utilized the TCAV protocol, which is a specific method of setting the airway pressure release ventilation (APRV) mode. A continuous positive airway pressure (CPAP) or inspiratory time (T_High_) is adjusted to approximately 90% of each respiratory cycle. The CPAP phase pressure (P_High_) is set sufficient to recruit collapsed lung tissue and regain functional residual capacity. The expiratory time (T_Low_) or release phase is set using the slope of the expiratory flow curve (Slope_EF_). Expiratory flow is stopped, and the lung is reinflated using a ratio of peak expiratory flow (E_PF_) to the point of expiratory flow termination (E_FT_) of 75% (i.e., E_FT_/E_PF_ = 0.75). Lastly, P_Low_ is set to 0 cmH_2_O, which allows minimal impedance of expiratory flow to remove CO_2_ during the brief release phase and accurate assessment of lung compliance using the Slope_EF_. However, the end-expiratory pressure never reaches 0 since the T_Low_ is set sufficiently brief to maintain a positive end-expiratory pressure, which we term time-controlled PEEP (TC-PEEP) (Fig. 2) [8, 9]. Groups 2 and 3 utilized a modified TCAV protocol, with all of the aforementioned TCAV settings except for P_Low_ of 5 cmH_2_O (group 2) and P_Low_ of 10 cmH_2_O (group 3). For groups 4–6, volume assist control (VAC) was used with the Acute Respiratory Distress Syndrome Network (ARDSnet) protocol, which uses LTV ventilation with a progressive PEEP scale.

**Table 1:** Ventilation groups and setting for the TCAV groups 1–3

Group no. Mode of ventilation (*n* = 10 runs/group)	E_FT_/E_PF_	P_High_ (cmH_2_O)	P_Low_ (cmH_2_O)	T_High_ (sec)	T_Low_ (sec)
1. TCAV protocol group 1 [8]	0.75	25	0	4.5	0.5–0.6
2. TCAV protocol group 2	0.75	25	5	4.5	0.42
3. TCAV protocol group 3	0.75	25	10	4.5	0.42–0.5

The progressive P_Low_ scale was employed to generate different combinations of I_PF_ and E_PF_

**Table 2:** Ventilation settings for the ARDSnet groups 4–6

Group no. Mode of ventilation (*n* = 10 runs/group)	I:E	V_T_ (cc)	T_i_ (sec)	RR (breaths/min)	Flow (L/min)	PEEP (cmH_2_O)
4. ARDSnet protocol group 4	1:2	240	1.6	12	40	0
5. ARDSnet protocol group 5	1:2	240	1.6	12	40	10
6. ARDSnet protocol group 6	1:2	240	1.6	12	40	20

Tidal volumes of 240 cc were chosen based on ARDSnet protocol; the standard 6 cc/kg was used in a calculation for a standard 40 kg pig. PEEP of 0, 10, and 20 cmH_2_O were chosen to reflect a progressive PEEP scale advocated by the ARDSnet protocol [11]. A maximum PEEP of 20cmH_2_O was chosen because this value generated a plateau pressure of ~ 25 cmH_2_O, most similar to the plateau pressure seen in the TCAV groups

Mucus movement was assessed by measuring the difference in centimeters between the proximal edge before and after ventilation (Fig. 1c). The primary variables that were recorded from the ventilator were peak inspiratory flow and peak expiratory flow (I_PF_ and E_PF_, respectively); also recorded were peak inspiratory pressure, plateau pressure, mean airway pressure, positive end-expiratory pressure (PEEP), minute ventilation, and tidal volume. After each run, the ETT was removed and cleaned, and the porcine lungs were cleaned with saline and suctioning to clean the respiratory tree of retained simulated mucus.

Mechanical ventilation was randomized into 6 groups (*n* = 10 runs/group) with settings defined in Tables 1 and 2. Some of the results of this study were previously published in abstract form.

### Statistical analysis

For each primary variable measured (proximal mucus movement, I_PF_, E_PF_), data are reported as a mean with standard deviation. To compare variables between groups, a paired sample *t* test was employed. A *p* < 0.05 was considered significant. Statistical analysis was performed using JMP10.

## Results

### Mucus clearance

**Fig. 3:**
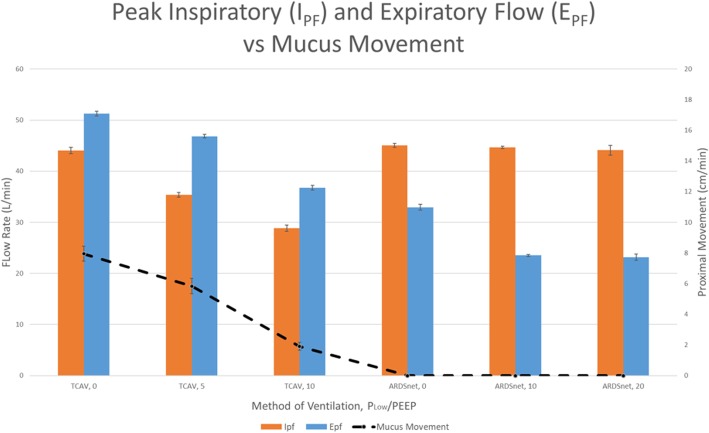
Combined bar/line graph comparing the peak inspiratory flow (I_PF_), peak expiratory flow (E_PF_), and proximal mucus movement for the different experimental groups. The I_PF_ and E_PF_ are indicated by the orange and blue colored bars, respectively, and correspond to the left vertical axis. Proximal mucus movement is indicated by the data points connected by the dotted line and corresponds to the right vertical axis. TCAV protocol groups 1, 2, and 3 utilized APRV with varying P_Low_ settings of 0 cmH_2_O (standard TCAV), 5 cmH_2_O, and 10 cmH_2_O, respectively. ARDSnet protocol groups 4, 5, and 6 utilized LTV with varying PEEP settings of 0 cmH_2_O, 10 cmH_2_O, and 20 cmH_2_O, respectively

In all three TCAV groups, proximal mucus movement was measured. Proximal mucus movement was greatest with the standard TCAV protocol (group 1, 7.95 ± 0.49 cm/min), followed by modified TCAV with a P_Low_ 5 cmH_2_O (group 2, 5.83 ± 0.50 cm/min) and modified TCAV P_Low_ 10 cmH_2_O (group 3, 1.91 ± 0.25 cm/min) (*p* < 0.05 for all groups). There was no proximal mucus movement in any of the three ARDSnet protocol groups (0 ± 0 cm/min) (Table 3; Fig. 3).

**Table 3:** Primary and secondary outcomes recorded

	TCAV, P_Low_ 0 (standard)	TCAV, P_Low_ 5	TCAV, P_Low_ 10	LTV, PEEP 0	LTV, PEEP 10	LTV, PEEP 20
Mucus movement (cm/min)	7.95 ± 0.49	5.83 ± 0.50	1.91 ± 0.25	0 ± 0	0 ± 0	0 ± 0
I_PF_ (L/min)	44.04 ± 0.63	35.37 ± 0.48	28.88 ± 0.62	45.06 ± 0.42	44.68 ± 0.23	44.11 ± 0.96
E_PF_ (L/min)	51.27 ± 0.45	46.84 ± 0.35	36.76 ± 0.44	32.94 ± 0.58	23.52 ± 0.21	23.16 ± 0.61
PIP (cmH_2_O)	25 ± 0	25 ± 0	25 ± 0	23.4 ± 0.72	26 ± 0.54	37.9 ± 0.23
P_Plat_ (cmH_2_O)	24.9 ± 0.1	24.9 ± 0.1	25 ± 0	10.08 ± 0.10	15 ± 0	26.81 ± 0.7
P_Mean_ (cmH_2_O)	21.8 ± 0.13	22.8 ± 0.13	23 ± 0	4.45 ± 0.05	12.7 ± 0.15	23.4 ± 0.16
PEEP (cmH_2_O)	N/A	N/A	N/A	0.57 ± 0.02	10 ± 0	20.2 ± 0.2
MV (L/min)	6.28 ± 0.15	4.01 ± 0.8	3.63 ± 0.12	3.01 ± 0.02	3.08 ± 0.03	3.13 ± 0.08
V_T_ (cc)	491.6 ± 11.61	315.5 ± 3.15	271.56 ± 5.91	243.4 ± 1.65	245.3 ± 1.45	241.2 ± 0.36

All values are reported in mean ± SE. *TCAV* time-controlled adaptive ventilation, *LTV* low tidal volume, *I*_*PF*_ peak inspiratory flow, *E*_*PF*_ peak expiratory flow, *PIP* peak inspiratory pressure, *P*_*Plat*_ plateau pressure, *P*_*Mean*_ mean airway pressure, *PEEP* positive end-expiratory pressure, *MV* minute ventilation, *V*_*T*_ tidal volume. Proximal mucus movement was significantly different between all groups (*p* < 0.05), except ARDSnet groups 4, 5, and 6. E_PF_ was significantly different between all groups (*p* < 0.05), except ARDSnet groups 5 and 6. Regarding I_PF_, there was no significant difference between TCAV group 1 and ARDSnet groups 4, 5, and 6; however, groups 1, 4, 5, and 6 had significantly greater I_PF_ than TCAV groups 2 and 3 (*p* < 0.05)

### Peak flows

The absolute values of the E_PF_ were significantly greater than those of the I_PF_ for each of the TCAV groups (*p* < 0.05). In contrast, the I_PF_s were significantly greater than the E_PF_s for each of the LTV groups (*p* < 0.05) (Fig. 3). APRV set with the standard TCAV protocol (group 1, P_Low_ 0) generated the highest E_PF_ of all TCAV groups (51.27 ± 0.45 L/min). As the P_Low_ was increased in the modified TCAV modes (groups 2 and 3), the E_PF_ and I_PF_ decreased. For the ARDSnet groups, the E_PF_ decreased as PEEP was increased from PEEP 0 (group 4, 32.94 ± 0.58 L/min) to PEEP 10 (group 5, 23.52 ± 0.21 L/min), but plateaued after that with PEEP 20 (group 6, 23.16 ± 0.61 L/min). There was no significant difference in I_PF_ between all ARDSnet groups and the TCAV group 1 (*p* = 0.61) (Table 3, Fig. 3); however, the I_PF_ in the ARDSnet groups were significantly greater than that of the TCAV groups 2 and 3 (*p* < 0.05 for both).

### Mucus movement and E_PF_

**Fig. 4:**
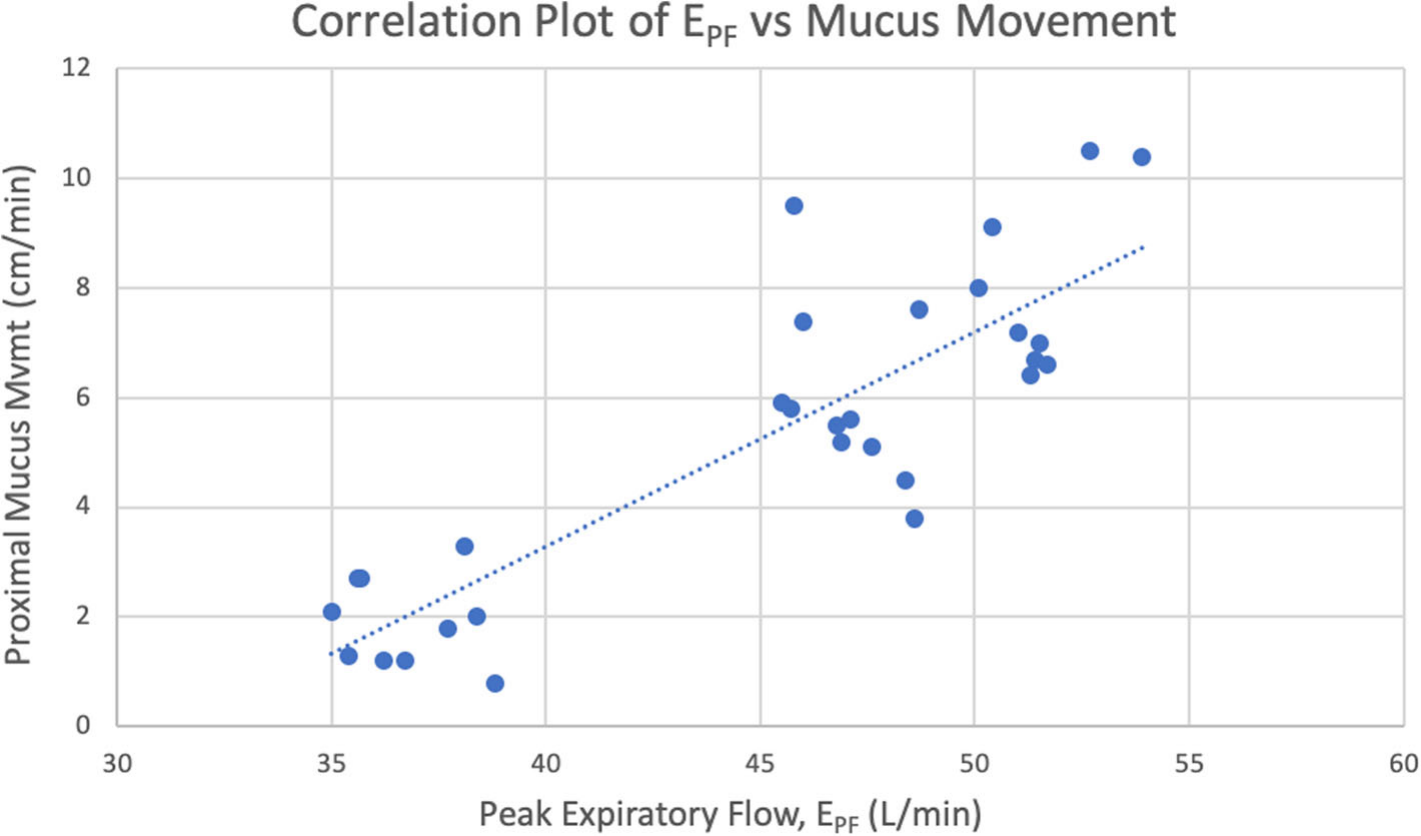
Scatterplot of E_PF_ and proximal mucus movement for all trials within the TCAV groups (*n* = 30) with a line of best fit, for which *R*^2^ = 0.74

Proximal mucus movement was most closely associated with the absolute value of E_PF_ in the TCAV groups, where E_PF_ > I_PF_. We found a strong positive correlation between E_PF_ and mucus movement among these groups (*R*^2^ = 0.74) (Fig. 4). There was no association between proximal mucus movement and E_PF_ in the ARDSnet groups, where I_PF_ > E_PF_.

## Discussion

The findings presented in this manuscript demonstrate that the TCAV protocol, using the APRV mode, resulted in proximal mucus clearance in our isolated lung model. If the TCAV protocol is also effective at facilitating mucus removal in patients, it could have important clinical ramifications. Treatment groups 4 through 6 were based on the ARDSnet protocol, which was chosen because it is the standard-of-care method for protective mechanical ventilation. Walkey et al. studied the incidence of VAP in ICU patients with pulmonary contusion and found that use of APRV was associated with less VAP, which they hypothesized was due to increased lung recruitment [15]. Although the TCAV protocol was not used in the Walkey et al. study, we showed that even if APRV was not set to the standard TCAV protocol (groups 2 and 3), there was still proximal mucus movement. Hence, we postulate that an additional or alternate mechanism for this clinical finding could be increased respiratory mucus clearance due to the high expiratory flows seen with the APRV mode. In fact, these effects might be additive. In the collapsed state, the lung cannot be cleared of mucus and is prone to pneumonia [16]. In the recruited lung, the open airways permit mucus movement and decrease the risk of pneumonia. Hence, the increased lung recruitment and postulated mucus clearance characteristic of APRV may work together to decrease the incidence of VAP.

Our data suggests that the combination of time and pressure parameters of the TCAV protocol effectively generates high expiratory flows. This protocol recruits the lung with each breath cycle, creating greater elastic recoil forces and increasing potential energy. This is then liberated as kinetic energy during the pressure release phase. During the brief release phase, lung recoil generates the relatively high expiratory flow rates measured in this study. In contrast, the ARDSnet protocol delivers a low tidal volume to the lung over a short inspiratory duration, which does not generate the same degree of pressure or elastic energy within the lung and leads to a significantly lower expiratory flow rate. It is due to this concept that the TCAV groups with increased P_Low_ (groups 2 and 3) still showed a significantly greater expiratory to inspiratory flow ratio; although there was some impedance to expiratory flow, the expiratory flow rate was still greater than inspiratory flow rate.

ARDSnet group 6 generated a plateau pressure (P_Plat_, 26.81 ± 0.07 cmH_2_O) most similar to the TCAV groups (25 ± 0.0cmH_2_O); however, the time spent at this pressure is different between the two protocols. The TCAV protocol’s increased time at plateau pressure produces a more complete recruitment with an increase in alveolar number rather than size [12]. As lung volume increases, airways are tethered open which lowers airway resistance to gas flow [13]. In other words, the higher mean airway pressures “stent” open the lungs, which lowers expiratory resistance and allows for greater peak expiratory flows. While ARDSnet group 6 generated a similar plateau pressure to the TCAV groups, the relatively short time at inspiration did not allow pressure or energy to build in the lung in the same manner, which is evidenced by the net flow in the inspiratory direction seen with ARDSnet.

Notably, the higher plateau pressures that facilitate lung recruitment in TCAV have not been associated with an increased risk of ventilator-induced lung injury (VILI). Indeed, previous studies using TCAV have shown that the extended time at P_Plat_ has the beneficial effect of redistributing gas to the alveoli from the alveolar ducts and promoting homogenous alveolar ventilation, rather than overdistending alveoli and causing VILI [8, 9]. The extended time at T_High_/P_High_ gradually recruits alveolar units in a non-pathologic manner, leading to increased lung volume and reduced strain at the alveolar level, despite the higher P_Plat_ [12, 13, 17]. A systematic review has shown that preemptive application of TCAV on trauma patients demonstrated a reduced ARDS incidence and mortality [18].

The TCAV groups, especially the standard protocol group 1, demonstrated significant proximal mucus movement whereas the ARDSnet groups showed no effect on mucus movement. We believe this is due to the difference in flows generated by each ventilation mode. For the TCAV groups, the E_PF_s were significantly greater than the I_PF_s which resulted in a net flow in the expiratory direction; the opposite was true for the ARDSnet groups. Hence, we postulated that this net expiratory force at least partially explains the mechanism for proximal mucus clearance. We also observed a trend between increasing E_PF_ values and increasing proximal mucus movement for the groups with E_PF_ > I_PF_. Group 1 (standard TCAV protocol) had the greatest E_PF_ and the greatest proximal mucus movement, followed by group 2 and then group 3. This positive correlation is shown in Fig. 4 (*R*^2^ = 0.74). We found a greater I_PF_ than E_PF_ for all ARDSnet groups, which caused a net inspiratory force in the distal (toward the lungs) direction. We believe this force prevented proximal mucus movement. Although the study model did not allow distal mucus movement to be observed or measured, we postulate that the simulated mucus was driven into the lung due to the net flow in the inspiratory direction, among other variables.

One might expect the mucus movement to be influenced by the magnitude of the vector force in the expiratory direction, which is represented by the difference between E_PF_ and I_PF_. However, we found that, when the E_PF_ was greater than the I_PF_, mucus movement correlated much more with the absolute value of the E_PF_ (*R*^2^ = 0.74, Fig. 4) rather than with the magnitude of the vector force in the expiratory direction (*R*^2^ = 0.002). This finding is supported by Kim et al., who also found that respiratory mucus movement was influenced most greatly by the absolute E_PF_ value rather than the difference between E_PF_ and I_PF_ [4]. Although the ARDSnet group 1 (PEEP 0) generated an E_PF_ value similar to TCAV group 3 (P_Low_ 10) (32.94 vs. 36.76 L/min, respectively), the ARDSnet group still did not generate any proximal mucus movement. We believe this is because the I_PF_ was greater than the E_PF_ in ARDSnet group, instead of vice versa, which creates a net force in the inspiratory direction.

There are limitations to our study. Due to IACUC regulations of minimal animal use, an ex vivo porcine lung model was used to obtain the data for this study. Accordingly, we cannot be sure how our results will translate to an in vivo or clinical setting due to variables missing from our study, such as chest wall resistance. Due to the chest wall tendency to expand, it would be expected that this would provide a slight opposing force during the expiratory phase of the TCAV breath protocol. We postulate this might affect the magnitude of the E_PF_s and mucus movement that was observed. Though we found that E_PF_ (when E_PF_ > I_PF_) and mucus movement had the strongest correlation across variables, we cannot definitively conclude that E_PF_ is the primary mechanism for proximal mucus movement. Numerous other factors (mean airway pressure, minute ventilation, etc.) vary among the experiment groups, and their effect on mucus movement cannot clearly be quantified. However, we do conclude with reasonable certainty that E_PF_ (when E_PF_ > I_PF_) accounts for at least a significant part of the results for proximal mucus movement seen here. Another limitation is that we washed and reused the same set of porcine lungs for all experiments. Any retained mucus beyond reach of the standard cleanout, or perhaps the cleanout process itself, could have altered the distal airways and impacted the later trials. Our model also limited us to measuring mucus distance within the ETT; it did not allow us to see how mucus moved within the bronchi and beyond. Applied clinically, much of the mucus driven by the TCAV protocol from the distal airways to the ETT might get stuck around the cuff of the ETT. This could theoretically be remedied with suctioning just beyond the tip of the ETT. Additionally, the ventilation mode alone would not be able to clear secretions that get stuck on the proximal side of the ETT cuff. These subglottic secretions are outside of the ETT and therefore not exposed to the ventilatory flows. However, subglottic secretion drainage methods already used clinically could supplement our TCAV strategy to maximize secretion clearance.

In summary, we demonstrated that a ventilation strategy (TCAV protocol) could be used to move mucus proximally in an isolated lung model. We postulate that the mechanism of proximal mucus movement is a combination of absolute expiratory flow rate and the net force vector in the expiratory direction. The current standard-of-care protective ventilation strategy (ARDSnet protocol) did not result in proximal mucus movement.

## Conclusions

The TCAV protocol resulted in the greatest proximal movement of simulated mucus in this excised porcine pulmonary system model. Proximal mucus movement was most strongly correlated with the absolute E_PF_ when the net flow rate was in the expiratory direction (E_PF_ > I_PF_). The ARDSnet protocol did not move simulated mucus proximally, even when generating expiratory flows similar to those seen in one of the TCAV protocol groups. We postulate that the increased E_PF_ relative to I_PF_ generated in the TCAV protocol is the probable mechanism for the proximal mucus movement measured in this study. We further speculate that if the TCAV protocol is applied clinically and has the same impact on increased mucus clearance in patients, it may reduce the incidence, morbidity, and mortality associated with VAP.

## Abbreviations

APRV: Airway pressure release ventilation
E_FT_: expiratory flow termination
E_PF_: Peak expiratory flow
ETT: Endotracheal tube
I_PF_: Peak inspiratory flow
LTV: Low tidal volume
PEEP: Positive end-expiratory pressure
P_High_: Pressure at inspiration (in APRV)
P_Low_: Pressure at expiration (in APRV)
TCAV: Time-controlled adaptive ventilation
T_High_: Time at high pressure
T_Low_: Time at low pressure
VAC: volume assist control
VAP: Ventilator-associated pneumonia
